# Optical tweezers in biomedical research – progress and techniques

**DOI:** 10.25122/jml-2024-0316

**Published:** 2024-11

**Authors:** Dharm Singh Yadav, Tudor Savopol

**Affiliations:** 1 Biophysics and Cellular Biotechnology Department, Carol Davila University of Medicine and Pharmacy, Bucharest, Romania

**Keywords:** optical tweezers, cell manipulation, single molecule studies, cell stretching, membrane tethering, elastic properties of cells, DNA stretching, CCD, Charge-Coupled Device, AFM, Atomic Force Microscopy, dsDNA, Double-Stranded DNA, ODS, Optical DNA Supercoiling, RNAP, RNA Polymerase, IVF, In-Vitro Fertilization, RBC, Red Blood Cells, HOT, Holographic Optical Tweezers, SLM, Spatial Light Modulator, *E. Coli, Escherichia coli*

## Abstract

Optical tweezers, which leverage the forces exerted by radiation pressure, have emerged as a pivotal technique for precisely manipulating and analyzing microscopic particles. Since Arthur Ashkin's ground-breaking work in the 1970s and the subsequent development of the single-beam optical trap in 1986, the capabilities of optical tweezers have expanded significantly, enabling the intricate manipulation of biological specimens at the micro- and nanoscale. This review elucidates the foundational principles of optical trapping and their extensive applications in the biomedical sciences. The applications of optical tweezers in biomedicine are vast, ranging from the investigation of cellular mechanical properties, such as cell stretching, membrane elasticity, and stiffness, to single-molecule studies encompassing DNA and protein mechanics, protein-DNA interactions, molecular motor functions, and pathogen-host interactions. Advancement of optical tweezers in this field includes their integration with holography, fluorescence microscopy, microfluidics, and enhancements in force sensitivity and positional accuracy. These tools have profoundly impacted the study of cellular mechanics, drug discovery processes, and disease diagnostics, providing unparalleled insights into the biophysical mechanisms underlying health and pathology.

## INTRODUCTION: OPTICAL TWEEZERS

Optical tweezers, a novel tool developed based on the concepts of radiation pressure, utilize the force exerted by light to trap and precisely manipulate tiny particles [1]. The fundamental study of radiation pressure and its applications in manipulating small particles began in the 1970s by Arthur Ashkin [2]. Later in 1986, Ashkin *et al*. [3] demonstrated a ‘single beam optical trap’ or ‘optical tweezers’, a versatile and highly efficient tool, significantly advancing the manipulation and analysis of microscopic objects. The optical tweezers utilize forces exerted by a tightly focused laser beam to capture and move small objects in three dimensions. By the 1990s, Ashkin *et al*. pioneered using optical tweezers to manipulate biological specimens, starting with a single tobacco mosaic virus and an *Escherichia coli* (*E. coli*) bacterium [4]. In 1993, Ghislain *et al*. introduced photonic force microscopy, which utilizes an optically trapped particle as a highly sensitive cantilever for probing microscopic force fields, detecting forces as small as femto-newtons (10^−15^N) to piconewtons (10^−12^N) [5]. Since the 1990s, optical force spectroscopy has been employed to study the mechanical properties of biomolecules and biological motors [6,7], which makes optical tweezers a crucial tool to manipulate single biological cells, biomolecules, bacteria, and viruses, providing non-contact force for manipulation with force resolution as accurate as 0.1 pN [8,9].

From the biomedical research perspective, optical tweezers are crucial for manipulating and exploring biological objects and processes at the micro- and nanoscale [10]. Their accuracy and versatility enable them to manipulate various biological samples, allowing them to study individual cells, subcellular components, and macromolecules, such as proteins, DNA, and molecular motors [11–13]. Optical tweezers have transformed the study of cellular mechanics by exerting precise forces on biological specimens, allowing researchers to elucidate the mechanical properties of cells, investigate the dynamics of cellular components, and unravel the complexities of cell-cell and cell-environment interactions [14]. Their application extends to drug discovery and development, where optical tweezers provide insights into the mechanisms of action of pharmaceutical compounds and facilitate the characterization of drug-target interactions [15]. Furthermore, optical tweezers are used in biomedical diagnostics and treatments, where they may manipulate and sort cells for disease diagnosis, drug administration, and regenerative medicine [8]. Their integration with other cutting-edge technologies and improved imaging modalities expands their utility, allowing for complex investigations and real-time monitoring of biological events [16–18]. In summary, optical tweezers have become essential tools in biomedical research, advancing our understanding of biological systems and opening new opportunities for medical innovation and therapeutic development.

### Optical trapping concept

The particles trapped by optical tweezers experience two types of forces known as scattering and gradient force [3]. The gradient force arises from the spatial light gradient, whereas the scattering force is generated by photons hitting the particle along their propagation direction. The gradient and scattering forces arise separately from the refraction and reflection of light. The refraction of incident light by a sphere alters the momentum of the light, causing an equal and opposite momentum change in the sphere due to momentum conservation.

Theoretical force calculations in optical tweezers are based on comparing the particle size and the wavelength of the trapping laser. The relationship between the particle’s diameter (d) and the laser beam’s wavelength (λ) is used to determine the forces acting on a particle, which are generally divided into three regimes: d >> λ, d << λ, and d ~ λ [19,20]. The theory of optical tweezers is complex, and only two simplified theoretical models have been developed to calculate the forces on particles: Rayleigh approximation and geometric optics or Mie theory [21]. The Rayleigh regime condition is applicable when the particle size is much smaller than the laser beam’s wavelength. Conversely, when the particle size is comparable to or larger than the wavelength of the laser beam, the particle behavior is better described by Mie theory or geometrical optics. The dipole approximation, which falls within the Rayleigh regime, is often used in the context of optical tweezers for trapping small particles, especially in biological applications [22]. The forces in optical traps depend on the size, refractive indices of the trapped object and surrounding medium, the laser beam gradient, and the laser power [23]. In the dipole approximation, it is shown that the trapping (gradient) force is given by the following formula [9,24]:


$${\vec{F}}_{OT}=\frac{2\pi r^{3}n_{1}}{c}\left(\frac{m^{2}-1}{m^{2}+2}\right)\vec{\nabla }I$$

where, *n_1_* is the absolute refractive index of the suspension medium, *c* is the velocity of light, *m* is the relative refractive index of the particle with respect to the medium and is the gradient of the laser beam intensity. If *m*>1 (the particle has a higher refractive index than the medium), has the same orientation as and the particle is pulled toward higher light-intensity regions (the focal point of the microscope lens).

**Figure 1:**
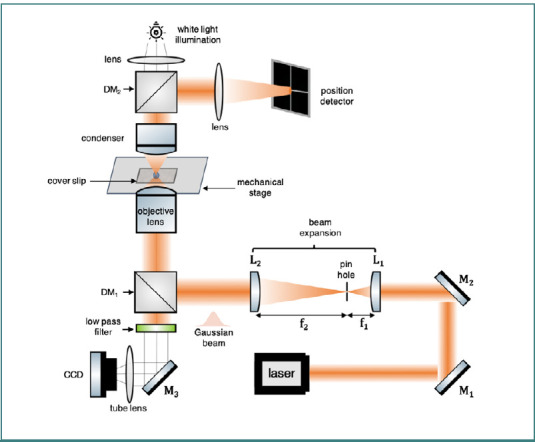
Schematic of a single beam optical tweezers system. An expanded Gaussian laser beam (usually an infrared laser for biological applications) is focused on the sample plane using a high numerical aperture microscopic objective lens to trap and manipulate a suspended particle in the focal point. The position detector analyzes the scattered light from the particle, and the corresponding optical trapping force is calculated. A CCD camera is used to capture the illuminated light passing through the sample to observe the trapping, and both the camera and position detector are controlled using a computer.

DM, Dichroic Mirror; M, Mirror; F, Focal Length; L, Lens.

From an experimental perspective, several methods have been developed to calibrate optical tweezers [25]. A typical optical tweezers system consists of several elements, including the laser source, beam expansion, steering optics, microscope with a high numerical aperture objective, fluidic chamber, charge-coupled device (CCD) camera, particle position sensing system (such as quadrant photodiode or QPD) and computer for recording and data analysis (Figure 1).

### Advanced optical tweezers

Recent advances in optical tweezers have broadened their applications in biology, where they are employed to explore biochemical and biophysical processes. Advanced optical tweezers have numerous optical traps, acousto-optic trap steering, and back focal plane interferometry position detection, which can detect sub-nanometer displacements and forces less than 0.1 pN [26]. Their compatibility with microscopy makes them ideal for lab-on-chip systems, allowing optical manipulation, actuation, and sensing [27]. Developments continue to improve instruments and methodologies by combining optical tweezers with other methods of manipulating or detecting single molecules [28].

Modern biomedical applications require innovative techniques for analyzing, sorting, and manipulating cells and biomolecules within the framework of a microfluidic chip. Optical tweezer setups are highly versatile and dynamic, allowing for the integration of various techniques. Their compatibility with liquid medium conditions has been used in transferring materials into single cells, cell sorting in microfluidic systems, and nanomechanical characterization of biological cells [8]. Advancements in optical trapping setups include the integration of methods such as holography [16], fluorescence microscopy [29], and microfluidics [30–32], which enhance the capabilities of optical tweezers, enabling more precise manipulation and analysis of biological systems at the molecular and cellular levels.

The incorporation of spatial light modulators (SLMs) and computation has evolved optical tweezers into holographic optical tweezers (HOT), capable of trapping and moving many objects at once [16,27]. This is particularly useful in assembling microscopic structures, studying colloidal systems, and high-throughput biological assays. HOTs have been effectively used in cellular studies, especially for sorting and analyzing multiple cells simultaneously [33]. Recent studies have highlighted the holographic optical tweezers to create and stabilize three-dimensional multicellular structures from embryonic stem cells, and the development of complex co-culture micro-environmental analogs that mimic adult stem cell niches, enabling precise manipulation of cellular microenvironments [34]. Moreover, HOTs are critical in three-dimensional manipulation, enabling optical tweezers to operate in environments such as lab-on-chip systems and microfluidics, where precise control of particle positioning is necessary. The ability to generate multiple optical traps increases throughput and reduces time, which is crucial for dynamic biological systems requiring high efficiency.

The coupling of optical tweezers with fluorescence microscopy offers real-time visualization of biological processes at the molecular level. This combination allows the manipulation and tracking of single molecules while observing their behavior using fluorescence markers [29]. Such integration is powerful in studying DNA-protein interactions, folding, and enzyme mechanics. For instance, studies combining optical tweezers, fluorescence microscopy, and microfluidics demonstrated the ability to directly observe and measure structural properties of DNA and DNA-protein complexes, molecular mechanisms of nucleo-protein filament assembly on DNA and the motion of DNA-bound proteins, and the interactions between DNA and transcription factors [35,36]. One of the significant applications is to investigate protein folding mechanisms and its misfolding [37], a key factor in various diseases such as Alzheimer’s and Parkinson’s. Applying controlled forces to the protein molecules allows the folding or unfolding of the protein to be induced while observing the changes through fluorescence, offering insights into molecular pathologies and potential therapeutic targets.

When combined with optical tweezers, microfluidics offers unparalleled precision in manipulating cells and biomolecules in controlled environments [32]. Microfluidic platforms enable the precise handling of small volumes of fluids, providing an optimal environment for biological experiments. Their integration with optical tweezers is advantageous in various applications such as single-cell analysis, cell sorting, and drug testing [38,39]. Optical tweezers within microfluidic systems can manipulate cells extremely accurately, allowing for detailed studies on cell mechanics, motility, and intercellular interactions [40]. For example, recent studies combining microfluidics with optical tweezers have shown that they trap and analyze red blood cells to investigate their mechanical properties under various conditions [41,42], which has implications for studying diseases like malaria or sickle cell anemia. Microfluidic integration is also crucial for developing lab-on-chip devices, where optical tweezers perform tasks, such as trapping and sorting in a miniaturized system [43].

In summary, optical tweezers can play a crucial role in various biomedical diagnostics when combined with various techniques. The dynamic optical tweezer setups, which integrate various techniques, enable the development of application-specific, highly efficient systems to sort and identify pathological cells from healthy ones and investigate complex molecular processes based on mechanical properties.

## BIOMEDICAL APPLICATIONS OF OPTICAL TWEEZERS

### Mechanical properties of cells

The mechanical properties of cells, such as membrane elasticity, stiffness, and cell interactions, can be analyzed using a trapped bead manipulated by optical tweezers. Using this method, the mechanical response of the attached cell component (usually cell membrane) is accessed, and its response can be recorded in various directions and at different loading rates to understand its viscoelastic behavior thoroughly, thereby allowing a more comprehensive understanding of cellular responses to mechanical stimuli and giving insights into fundamental biological processes underlying disease development. Indentation (deformation), cell stretching, and tether extension are commonly used to access these properties [40]. Furthermore, by trapping intracellular organelles like lipid droplets, it is also possible to study the viscoelastic properties of the cell cytosol under different conditions [44].

### Cell indentation or deformation

**Figure 2:**
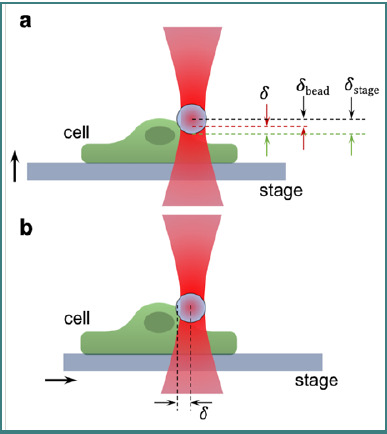
Examples of (A) axial and (B) lateral indentation; δ represents the indentation on the cells as a result of applied force using optical tweezers (figure re-created based on [49] under the terms of CCBY 3.0 license and contains icons from Reactome library under CCBY 4.0 license).

Indentation experiments using optical tweezers involve measuring the deformation of an attached cell caused by an external force using a trapped dielectric bead. As shown in Figure 2A, an axial indentation can be achieved either by moving the sample with a piezoelectric stage against the trapped dielectric bead [45] or by directing the trap towards the cell (in a linear [46] or oscillatory [47] manner). Another method involves lateral deformation (Figure 2B), where the sample or the trap is moved along the image plane against a perpendicularly positioned cell membrane [48].

The mechanical parameters of cells deduced from indentation data are usually obtained using the Hertz model [50–52]. The Hertz model regards the cell as a homogeneous elastic solid with shallow indentation depths, which allows for the exclusion of viscous components. As a result, the cell is defined by Young's modulus, which measures stiffness, and Poisson's ratio, which indicates compressibility [45]. Precise measurement of cell elasticity is crucial because it indicates cellular changes that can occur in various diseases. Although limited by cell homogeneity and elasticity assumptions, the Hertz model remains valuable for calculating cell stiffness. However, its accuracy depends on precise data from tools like optical tweezers. Optical tweezers offer significant advantages in this context compared to methods like atomic force microscopy (AFM) or micropipette aspiration. Unlike AFM or micropipette aspiration, which involves physical contact and may damage cells [53,54], optical tweezers are contactless and non-invasive, providing precise, three-dimensional force application with high spatiotemporal resolution. This precision allows for more accurate force-deformation data, making the combination of optical tweezers and the Hertz model highly effective for real-time, dynamic cell stiffness analysis.

The optical trapping-based approach has been employed on various cells, such as Balb3T3 cells, to evaluate their localized stiffness using two different-sized particles with optical tweezers and the Hertz model [46]. The measured cell stiffnesses were 17 Pa with 4 µm particles and 40 Pa with 2 µm particles. Coceano *et al*. investigated the mechanical properties of three human breast cell lines: normal myoepithelial (HBL-100), luminal breast cancer (MCF-7), and basal breast cancer (MDA-MB-231) using AFM and optical tweezers [45]. Their results showed that the MDA-MB-231 cells were significantly softer than the other two cell types in both methods. However, when analyzed by AFM and optical tweezers, HBL-100 and MCF-7 cells exhibited different behaviors. The study highlights the importance of using integrated approaches for mechanical phenotyping and suggests that cell stiffness measurements could provide insights into cancer progression and metastasis potential beyond molecular markers.

Yousafzai *et al*. proposed a method for cell indentation at pico-newton forces by moving a cell against a trapped microbead and calculating the elastic modulus using the Hertz model applied to HBL-100 cells [55]. Two characteristic regions for cell–bead interaction were described: indentation, when the stage/cell moves toward the bead, and retraction when the cell moves back. Their results showed that cells cultured on a bare substrate had an elastic modulus of 26 ± 9 Pa, which decreased to 19 ± 7 Pa on a collagen-coated substrate. During retraction, the values were 23 ± 10 Pa and 13 ± 7 Pa, respectively. These results demonstrate that cells adjust their stiffness to the substrate, showing the method's potential for probing cell mechanics influenced by the environment.

Furthermore, Zhou *et al*. [48] presented a rate-jump indentation method using optical tweezers to measure the stiffness of highly soft, non-adherent blood cells, like K562 myelogenous leukemia cells. They addressed the challenges of rate-dependent results in constant-rate loading tests by providing an invariant elastic modulus. The method, applying pico-newton forces, accurately measures the intrinsic elastic modulus of these cells. Additionally, it effectively differentiates the stiffness of myelogenous leukemia cell lines (K562 and HL60) from normal leukocytes, suggesting its potential for identifying diseased cells from normal ones without biochemical analysis.

### Cell stretching and membrane tethering

**Figure 3:**
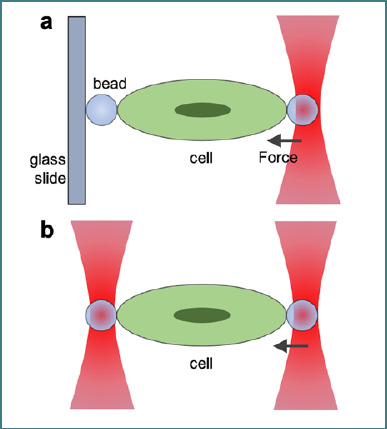
Schematic showing stretching of a cell using (A) single optical trap and (B) dual optical traps. The beads are coated using specific linkages supporting the suitable experiments.

Optical tweezers stretch cells and subcellular components, thus allowing the study of their elastic and viscoelastic properties. It can be achieved by single-trap [56,57] or dual-trap [58] setup, as depicted in Figure 3. In a single trap setup, two beads are positioned on opposite sides of the cell, out of which one bead is fixed to a stationary glass slide as a reference, while the other bead is trapped and moved with optical tweezers to stretch the cell and force measurement (Figure 3A). In the dual-trap optical tweezer configuration, one bead is held by a trap, and another trap moves the second bead to create tension in the cell (Figure 3B). Alternatively, a dual-trap configuration without beads can be used by directly focusing the laser beams on the cell [59].

The beads used in these experiments can be coated with various substances to enhance functionality and compatibility with biological samples. Functionalized beads can interact with specific cell membrane sites, aiding targeted studies [60]. For example, protein-coated beads facilitate cell attachment by mimicking the extracellular matrix [61]; Poly-L-lysine-coated beads increase cell attachment by enhancing the positive surface charge [62]; Biotin-coated beads create solid and specific interactions with biotinylated molecules [63], and PEG-coated beads reduce nonspecific interactions and prevent protein adsorption [64].

Using image or video processing, the induced geometric changes in the cell can be analyzed using a mechanical model describing the cell shape and deformations to extract the elastic or viscoelastic properties of the cells. Using this method, a number of studies have accessed the reduced bending stiffness of membrane skeletons in red blood cell (RBC) ghosts compared to intact membranes and derived elastic constants from force-extension curves after disrupting the skeletal network [65–67]. Tan *et al*. have reported that differentiated human embryonic stem cell-derived cardiomyocytes (hESC-CM) have a higher elastic modulus and viscosity than undifferentiated hESC, indicating improved cellular organization post-differentiation due to the loss of pluripotency [68].

**Figure 4:**
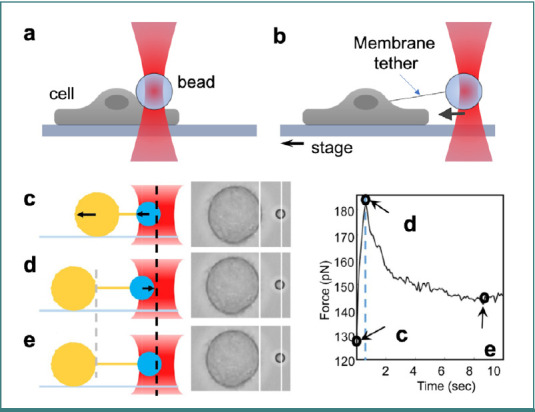
Illustration of cell membrane tethering using optical tweezers. A, an optically trapped bead pushed against an attached cell membrane results in (B) a spontaneous membrane tether formation. By applying controlled forces on the trapped bead using optical tweezers and moving the stage, the membrane's mechanical properties can be accessed. C-E, an example of a quantitative assessment of dynamic membrane mechanosensation in HeLa cells. C, the HeLa cell (shown as a large yellow circle) moves to the left, causing the membrane tether to stretch. The polystyrene bead (small blue circle), held by optical tweezers, is pulled leftward as the tension increases. The line between the circles depicts the membrane tether, and the arrows show the direction of movement. D, when the cell stops, the bead is drawn back toward the center of the optical tweezers. E, the bead remains still once the tension reduces to static tension; the right side of the graph illustrates the tether tension relaxation over time, with black circles indicating the points from C to E (figure (A-B) contains items from Reactome icon library under CCBY license, and (C-E) adapted from [69] under the terms of the CCBY 4.0 license).

A cell membrane tether is a spontaneously generated thin, cylindrical extension of the membrane, which can be created by attaching tiny beads to cell membranes and applying controlled forces via optical tweezers (Figure 4A and B). Beads used for membrane tethering experiments are typically coated with biotin, antibodies, ligands, lectins, streptavidin, fibronectin, or other functional molecules to enable specific interactions with membrane components or proteins [40]. An experimental example quantifying membrane mechanosensation in HeLa cells through tether tension dynamics is illustrated in Figure 4C-E [69]. As the HeLa cell moves, it stretches a membrane tether connected to a bead held by optical tweezers (Figure 4C). When the cell stops moving (Figure 4D), the bead returns to the center as the tension in the tether relaxes. Once equilibrium is reached, the bead remains stationary, indicating a state of static tension (Figure 4E). The accompanying graph illustrates the reduction in tether tension over time, highlighting key moments when tension fluctuates. This setup provides precise measurements of how cells mechanically respond to environmental forces.

This method can elucidate how cells deform and respond to external forces, a crucial aspect of various biological processes, including cell division, migration, and environmental interaction [70–72]. Using membrane mechanical models, the membrane mechanical properties can be determined using the force-elongation curves obtained from the tethering experiment with optical tweezers [73–76]. Darius V. Köster reported extracting lipid membrane tethers from cell plasma membranes using the optical tweezers-based membrane tether extension method to measure membrane tension and probe membrane reservoirs, providing insights into cell membrane mechanics and tension regulation during mechanical or chemical perturbations [77]. Pradhan *et al*. compared the mechanical properties of MCF7 cells with heterochromatin protein 1α knockdown (MCF7 HP1α KD) and MCF7 control breast cancer cells [78]. The membrane tension and force relaxation curves for these cells were measured, revealing that depletion of HP1α KD from MCF7 cells results in a decreased membrane tension compared to control MCF7 cells, an indication of increased malleability of these cells. This reduction in membrane tension suggests that HP1α plays a critical role in maintaining the mechanical integrity of the cell, affecting both the nuclear and plasma membranes.

The mechanical properties of cells, including membrane elasticity, stiffness, and interactions, are crucial for understanding cellular responses to mechanical stimuli and the underlying biological processes in disease development. In conclusion, methods such as indentation, cell stretching, and membrane tethering using optical tweezers provide valuable insights into these properties. Studies on different cell types, including HP1α-depleted MCF7 breast cancer cells, have highlighted significant differences in mechanical properties, underscoring the importance of mechanical analysis in cell biology and disease research. For a brief overview of studying the mechanical properties of cells using optical tweezers, we recommend a dedicated review article by Arbore *et al*. [40].

### Single-molecule studies

#### DNA mechanics

Optical tweezers have become an imperative tool in studying DNA mechanics, providing detailed insights into the physical properties of DNA [79–81] and its interactions with various proteins [12,82,83]. By manipulating individual DNA molecules, optical tweezers allow the exploration of fundamental aspects of DNA structure, dynamics, and function. DNA stretching and unzipping are two key applications in this area.

**Figure 5:**
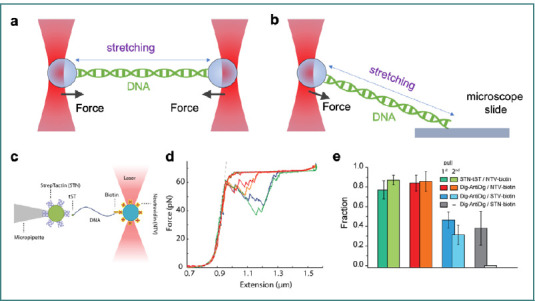
Illustration of DNA stretching methods and analysis of mechanical stability using optical tweezers. A, depiction of a dual optical trap system, where both ends of the dsDNA molecule are attached to optically trapped beads, allowing symmetric stretching. B, a single optical trap configuration with one DNA end attached to a bead held in the optical trap while the other end is anchored to a microscope slide. C-E, schematic and experimental example of the single optical trap method, where one end of dsDNA is attached to a bead in the optical trap, and the opposite end is held by a bead immobilized via a pipette. D, the force-extension curve reveals characteristic overstretching of dsDNA at ~65 pN, followed by stepwise relaxation, confirming mechanical stability. E, results comparing the mechanical resistance of various linkage strategies under 60 pN force. The STN-based system (STN-biotin-DNA-Dig(AntiDig)) demonstrates limited durability, as most tethers break during the first pull, highlighting potential challenges for its use in force spectroscopy applications (figure (C-E) adapted from [88] under the terms of the CCBY 4.0 license).

The stretching of a DNA molecule using optical tweezers allows us to investigate its elasticity and mechanical properties. In a typical experiment, one end of the DNA molecule is attached to a microbead trapped by optical tweezers, while the other end is fixed to another bead held by optical tweezers or a stationary surface (Figure 5A and B) or a micropipette (Figure 5C) [84]. By applying a controlled force to the bead, the DNA molecule can be stretched [85], unzipped [86], and twisted [87], and the measured resulting force can be used to study its elastic properties. Once DNA's elastic properties have been established, optical tweezers can be employed in various experimental settings to examine DNA processing enzymes, protein folding/unfolding, and protein-DNA interactions [13].

Figure 5C-E presents a schematic and experimental example using the single optical trap and pipette method. The force-extension curve (Figure 5D) shows the overstretching transition of dsDNA at ~65 pN, followed by a stepwise relaxation, validating the mechanical stability of the molecule. Figure 5E compares the mechanical resistance of different linkage strategies under 60 pN force. The STN-based system (STN-biotin-DNA-Dig(AntiDig)) exhibits limited mechanical durability, with most tethers breaking on the first pull, highlighting potential weaknesses for force spectroscopy applications [88].

A single DNA polymer (or molecule) behaves like a simple elastic spring when stretched slightly or moderately beyond its normal length. The elastic behavior of these springs is characterized by a property known as the persistence length, which quantifies the polymer's resistance to bending [89]. This persistence length is a fundamental mechanical property that reflects the stiffness of the polymer and is determined by the molecule's local elastic characteristics. Stretching studies using optical tweezers have provided significant insights into the elastic properties of DNA. For instance, Wang *et al*. reported that the persistence length of DNA is approximately 47 nm in a buffer containing 10 mM Na^+^ [85]. This value decreases to around 40 nm in the presence of multivalent cations like Mg^2+^ or spermidine, which shield the negative charges on the DNA backbone. Kandinov A, Raghunathan K, *et al*. showed that the elasticity of DNA is influenced by its sequence, with AT-rich sequences exhibiting a persistence length of 44 nm and CG-rich sequences showing 59 nm [90].

In this case, another important aspect of DNA is its contour length. The contour length of a polymer chain represents the maximum extension of the chain when fully stretched out, and the total number of monomers determines it, each contributing a fixed length (e.g., 0.338 nm per base pair for B-form DNA) [91]. When a molecule like double-stranded DNA (dsDNA) is stretched to its contour length, it reaches its full natural extension. If a force that exceeds this length is applied, the molecule undergoes what is known as an overstretching transition. This transition is a cooperative process where the DNA extends beyond its typical B-form configuration, leading to significant structural changes in response to the applied force. Using optical tweezers, Wenner *et al*. demonstrated the DNA overstretching transition depending on the salt concentration [92]. As the sodium ion concentration was increased from 2.57 mM to 1000 mM, the persistence length of DNA decreased from 59 nm to 46 nm, while the elastic stretch modulus remained approximately constant. Furthermore, Baumann *et al*. studied how trivalent cations can induce DNA condensation, which significantly alters its elastic response, showing behaviors such as a stick-release pattern or a plateau at approximately 20 pN [93]. These findings underscore the complex interplay between ionic conditions, DNA sequence, and molecular interactions in determining the elastic properties of DNA.

In typical DNA unzipping experiments using optical tweezers, one strand of a double-stranded DNA (dsDNA) molecule is attached to a trapped bead in dual optical tweezers, and a force is applied to separate the strands by pulling the beads apart. The DNA unzipping process mimics the action of helicases during DNA replication and transcription, where the double helix must be unwound to allow access to the genetic code. The force required to unzip the DNA provides insights into the stability of the double helix and the strength of the hydrogen bonds between complementary bases. By monitoring the force-extension curves during the unzipping of the strands, the sequence-dependent stability of DNA regions, binding sites of DNA-binding proteins, and the kinetics of DNA strand separation can be studied [95].

**Figure 6:**
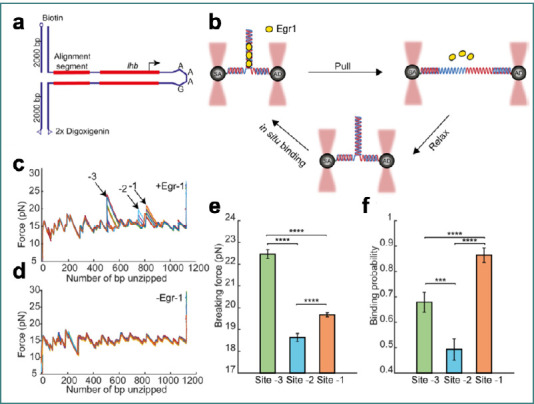
Single-molecule probing of Egr-1 binding to the Lhb promoter using optical tweezers highlights the role of optical tweezers in probing the mechanical stability of protein-DNA interactions and provides insights into the unzipping mechanism and binding affinity of Egr-1 to the Lhb promoter. A, schematic of the experimental construct, where the −517/+246 segment of mouse Lhb DNA is attached to a 350 bp alignment sequence and a stem-loop that stabilizes the tether after unzipping. B, the DNA construct is tethered between two polystyrene beads using 2-kb double-stranded DNA handles, with one bead trapped by optical tweezers to apply force. Egr-1 binding occurs in situ, followed by controlled unzipping and relaxation to study protein-DNA interactions. C, unzipping curve in the presence of Egr-1, revealing specific binding sites (-1, -2, -3) on the DNA marked by arrows. D, unzipping curve without Egr-1, demonstrating the distinct mechanical response without protein binding. E, Quantification of the breaking forces for Egr-1 at the three binding sites. F, Binding probability at the three sites, showing significant differences in affinity based on the number of unzipping cycles and protein concentration (figure reused from [94] under the terms of the CCBY-NC license).

A DNA unzipping experiment, as depicted in Figure 6, highlights the use of optical tweezers to study the mechanical stability and binding dynamics of Egr-1 to the Lhb promoter. By tethering the DNA construct between two beads and applying controlled force, the unzipping process reveals specific Egr-1 binding sites, offering insights into how protein-DNA interactions influence the mechanical behavior of the DNA. The experiment quantifies the binding forces and affinity at distinct sites, demonstrating how Egr-1 impacts the unzipping mechanism and providing valuable data on its role in transcription regulation. This approach allows for precise probing of protein-DNA interactions under mechanical stress [94].

The force signals observed during the unzipping and rezipping of DNA reveal characteristic flips between different values at specific positions, which are determined by the base sequence, indicating bistabilities in the position of the opening fork [86]. Additionally, double optical tweezers have enabled the visualization of protein-DNA interactions, such as the suppression of DNA unzipping by the tumor suppressor protein p53, which introduces an energy barrier to the unzipping process [86,96]. The statistical properties of metastable intermediates in DNA unzipping have also been characterized, showing a power-law distribution of unzipping region sizes, ranging from one base pair to over a hundred base pairs, with a significant fraction of small unzipping regions being undetected due to the high compliance of single-stranded DNA [97]. These findings underscore optical tweezers' high sensitivity and precision in studying the dynamic behavior of DNA and its interactions with proteins, providing valuable insights into fundamental biological processes such as DNA replication, repair, and transcription [82,98].

**Figure 7:**
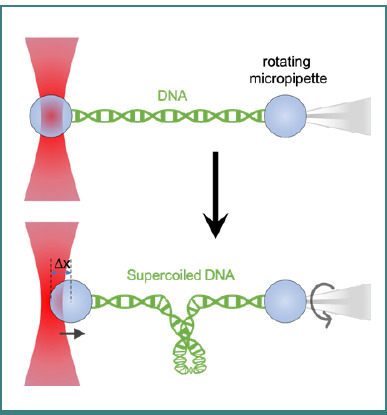
Simultaneous generation of supercoiled states of DNA and force measurement using single-trap optical tweezers and a rotating micropipette trap (figure re-created based on [99], Copyright (2003) National Academy of Sciences).

The Optical DNA Supercoiling (ODS) method using optical tweezers, which can be utilized to generate underwound (negatively supercoiled) DNA using single-trap optical tweezers and a rotating micropipette (as shown in Figure 7) [99] or dual-trap optical tweezers [100]. Torsional stress plays a vital role in many genomic processes, including replication and transcription, and often results in underwound DNA. Supercoiled DNA can exhibit different mechanical properties than relaxed DNA, affecting the force required for unzipping [101]. Additionally, optical tweezers integrated with fluorescence imaging can be used to study unique insights into the biomechanical properties of underwound DNA and the dynamics of DNA-protein interactions [102]. For instance, optical tweezers-based ODS experiments have revealed that the mitochondrial transcription factor A diffusion is hindered by local regions of underwound DNA, suggesting a regulatory mechanism for mitochondrial transcription [102]. The development of angular optical tweezers has facilitated the study of torque generation during transcription, offering detailed protocols for constructing and calibrating the instrument, preparing DNA templates, and analyzing real-time data [103]. These advancements underscore the versatility and precision of optical tweezers in elucidating the complex behaviors of DNA under torsional stress, thereby enhancing our understanding of fundamental genomic processes [87,104]. These studies contributed to our understanding of DNA replication and transcription regulation, where supercoiling plays a crucial role.

In recent years, optical tweezers have continued to advance the understanding of DNA mechanics, providing key insights into DNA structure, elasticity, and its interactions with proteins. Recent advances using dual-trap optical tweezers allow rapid torque control with spatial manipulation and fluorescence microscopy [102]. Using such systems, studies have also explored the role of negative supercoiling in genomic processes such as transcription regulation and genome stability, showing that negative DNA supercoiling significantly increases CRISPR-Cas9 off-target activity by inducing sequence-specific binding and enhancing mismatch tolerance during genome editing [101]. Furthermore, the integration of optical tweezers with fluorescence microscopy has allowed us to simultaneously manipulate and visualize DNA-protein interactions in real time, providing unprecedented insights into protein dynamics on DNA and chromatin, as well as uncovering force-dependent behaviors in complex DNA configurations [105]. These advancements highlight the crucial role of optical tweezers in exploring DNA mechanics and their increasing applicability in molecular biology.

Overall, using optical tweezers in DNA mechanics has provided profound insights into DNA's physical properties and behaviors under various conditions. It allows researchers to explore DNA's elasticity, stability, and interactions with proteins, enhancing our understanding of fundamental biological processes and the molecular basis of genetic regulation. These studies are essential for unraveling the complexities of DNA mechanics and its role in cellular functions and disease mechanisms.

#### Protein studies

Deciphering the complex dynamics of proteins and protein-DNA interactions is essential to understanding various disease processes. Optical tweezers have shown to be an effective scientific instrument in this endeavor. They allow us to interrogate the structure and folding of proteins in detail by precisely regulating their movements. This is critical for characterizing folding energy landscapes at high resolution, studying structurally complex multidomain proteins, folding in the presence of chaperones, and investigating real-time cotranslational folding of polypeptides [37].

**Figure 8:**
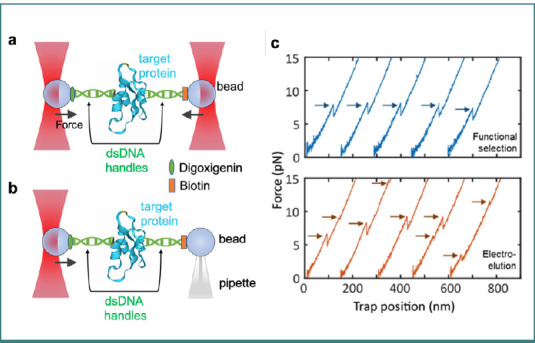
Schematic of experiment on protein folding kinetics using (A) dual optical trap and (B) single optical trap. The two ends of the target protein molecule are attached to two trapped beads using dsDNA handles using specific linkages, and by applying a controlled force on one end using the optical tweezers, the target protein can be unfolded, and its mechanical properties can be accessed based on the measured forces. C, a comparison of force-extension curves for the cAMP-binding domain A (CBD-A) of Protein Kinase A in its unliganded state using the single optical trap setup. The top panel shows curves for protein samples purified by functional selection, while the bottom panel displays curves for samples purified by electro-elution, highlighting differences in mechanical behavior based on the purification method (data shown in figure (C) adapted from [106] under the terms of the CCBY 4.0 license).

In a protein folding-unfolding experiment, a target protein is coupled to two microbeads via dsDNA handles. In this setup, the microbeads can be manipulated using either single-beam optical tweezers in combination with a trapping pipette (Figure 8A) or dual optical tweezers (Figure 8B). These configurations allow precise control over the position and forces applied to the trapped target protein. The trapped beads act as manipulators, enabling them to hold, steer, and apply tension to the protein, thereby facilitating the study of protein mechanics under varying force conditions. A comparison of force-extension curves for the cAMP-binding domain A (CBD-A) of Protein Kinase A in its unliganded state illustrates how different purification methods—functional selection versus electro-elution—affect the mechanical behavior of the protein (Figure 8C) [106].

**Figure 9:**
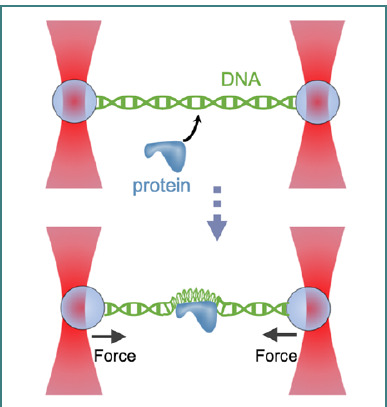
Schematic of a dual-trap optical tweezers setup applied to study protein-DNA interaction mechanics. The experiment can be conducted in either passive mode, where the trap separation remains constant, and both force and extension vary as a protein binds to and wraps the tethered DNA, or constant force mode, where the trap separation is adjusted to maintain a steady force, allowing changes in DNA extension while the force remains fixed. This setup demonstrates the flexibility in extracting force-extension data from protein-DNA interactions (Figure contains items from Reactome icon library under CCBY license).

In a different experimental approach to studying the protein DNA interactions, a DNA strand is tethered between two microbeads trapped by optical tweezers. When a protein molecule binds to the DNA and wraps around it, this binding causes changes in both the force exerted on the molecule and its end-to-end extension over time (Figure 9). This setup enables exploring protein-DNA interactions, including binding, unbinding, and structural changes, by applying controlled forces and observing the resulting molecular behavior.

DNA molecules intended for tethering experiments are typically functionalized with biotin and/or digoxigenin by incorporating modified bases or DNA primers, enabling them to bind to antibodies [106]. Proteins can then be tethered to these DNA handles through specific antibodies or affinity peptide tags (e.g., Avi or ybbR tag) [107]. Micron-sized beads coated with streptavidin or protein G are utilized to attach the molecule of interest, as they can bind to the antibodies. A common method involves forming a disulfide bond between a free cysteine in the protein and maleimide-functionalized DNA. Enzyme-based coupling methods also facilitate covalent protein-DNA linkages. In these methods, a fusion protein (e.g., HaloTag) or a peptide tag (e.g., ybbR tag) engineered into the protein undergoes an enzymatic reaction with a substrate cross-linked to DNA [13].

In this context, the optical tweezers were initially utilized to measure the force produced by a single *E. coli* RNA polymerase molecule during transcription [108]. Later, Block *et al*. employed this method to investigate the force-dependent behavior of RNA polymerase (RNAP) [109]. Their results provide new insight into the mechanism underlying force-induced stalling and challenge previous theories. In contrast to traditional motor proteins, where force acts directly to obstruct translocation, large forces seem to cause RNAP to travel significantly backward (5–10 base pairs), which may cause the protein to pause or reverse its direction along the DNA.

Recent advancements in combining optical tweezers with high-resolution fluorescence microscopy have significantly propelled the field of single-molecule biophysics [110–112]. This synergistic approach enables precise manipulation of individual molecules while offering detailed visualization and tracking of fluorescently labeled components. In DNA studies, this method facilitates real-time observation of interactions between DNA and various proteins, such as those involved in replication, transcription, and repair, while measuring the DNA molecules’ forces and mechanical properties [113]. For protein research, integrating optical tweezers and fluorescence microscopy allows for the in-depth study of protein folding, dynamics, and interactions with other biomolecules [12]. This dual technique provides profound insights into the molecular mechanisms underlying biological processes and the physical principles of biomolecular functions, thereby deepening our understanding of complex cellular phenomena and disease mechanisms.

Optical tweezers are useful tools to investigate disease etiology by examining how mutations modify proteins or protein-DNA binding and how environmental influences modify these interactions. This knowledge paves the way for developing targeted therapies and cures, bringing us closer to overcoming many diseases. Force-based investigations on proteins and DNA-protein complexes provide an extensive data set to elucidate the interplay between biochemical reactions and mechanical work. We recommend this review article by Bustamante *et al*. [13] for a detailed overview of the single-molecule studies of protein and DNA using optical tweezers.

### Molecular motors

**Figure 10:**
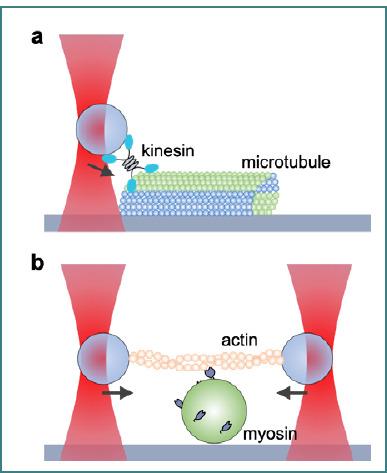
Schematic of (A) a single optical trap in measuring the force when a single kinesin motor interacts along a microtubule and (B) a dual optical trap to determine the mechanical results of ATP hydrolysis by a single myosin molecule.

In the field of molecular motor function research, optical tweezers offer a distinct advantage by allowing researchers to directly observe and manipulate the mechanical activities of motor proteins such as kinesin, dynein, and myosin [114–116], which are required for a variety of cellular processes such as intracellular transport, cell division, and muscular contraction. These motor proteins transform chemical energy from ATP hydrolysis into mechanical work while moving along cytoskeletal filaments in a highly regulated manner (kinesin and dynein move along microtubules, while myosin moves along actin filaments, see Figure 10A and B) [117].

By attaching a small bead to a motor protein or its substrate, optical tweezers can grasp and move it with nanoscale precision, allowing for a thorough investigation of the protein's movement and the forces it produces. One of the most important contributions of optical tweezers in this field is their capacity to monitor motor protein stepping behavior. For example, optical tweezer investigations demonstrated that kinesin moves in distinct 8-nanometer steps, corresponding to the distance between neighboring binding sites on a microtubule [118].

Optical tweezers have revealed crucial insights into the cooperative behavior of motor proteins, such as kinesin and dynein, by enabling the study of their interactions, force generation, and adaptive responses in living cells [119]. These investigations have demonstrated the strength and plasticity of motor protein systems in response to changing cellular environments. These findings improve our fundamental understanding of cellular mechanics and have potential applications in designing targeted therapeutics for diseases caused by motor protein dysfunctions.

### Cell manipulation and sorting

In cell manipulation and sorting, optical tweezers have revolutionized the ability to control and precisely analyze cellular behavior at the microscale. One key advantage of optical tweezers is that they enable contactless and label-free cell isolation and manipulation, which is crucial in minimizing potential damage and preserving cell integrity. Optical tweezers integrated with various other methods showed promising results in isolating single cells. For example, optical tweezers integrated with polydimethylsiloxane (or PDMS) devices enabled single yeast cell isolation with precise manipulation and high cell viability at low laser powers [120]. Using an optical tweezers method with photonic crystals and parylene-C films, Jing *et al*. measured anti-adhesive forces between human pluripotent stem cells and created patterned cell colonies, demonstrating effective cell manipulation and culturing with low laser intensity [121]. The development of optical tweezers has significantly advanced the study of RBC membrane mechanics, intercellular interactions, and cellular components, enhancing our understanding of RBC growth, development, and associated conditions [41]. Moreover, optical tweezers integrated with microfluidics, fluorescence, holography, and various other techniques were employed for sorting and purification of biological specimens, including 2 µm small mitochondria, yeast cells, rod-shaped bacteria, and 30 µm large protoplasts [38,122]. Advanced optical tweezers also enable nanomechanical characterizations of cells and transporting foreign materials into single cells, which are critical for stem cell delivery, tissue engineering, and regenerative medicine [123]. Their integration with robotic systems significantly enhanced control and efficiency for precise cell manipulation [124,125], facilitating applications such as cell migration studies, single-cell surgery, and preimplantation genetic diagnosis. In more recent studies, optical tweezers integrated with holography and dielectrophoresis are shown to perform automated and highly controlled cell sorting of circulating tumor cells from blood samples, drug-treated cancer cells with phenotypic heterogeneity for clinical applications, or fundamental studies [126–129].

The integration of optical tweezers with microfluidic systems, holography, and fluorescence techniques has significantly enhanced the precision and functionality of cell sorting technologies. Studies have demonstrated the efficacy of optofluidic chips for virus manipulation, achieving highly accurate nanoscale sorting and manipulation [130]. This approach leverages photonic crystal slabs and plasmonic enhancements to generate substantial optical forces while minimizing thermal effects, balancing efficacy and sample integrity [131]. As discussed previously, HOTs stand out for their ability to simultaneously manipulate multiple cells, providing unparalleled spatial control. This is particularly advantageous for complex sorting tasks, although it comes with increased cost and complexity [132]. Conversely, the incorporation of fluorescence allows for real-time identification and tracking of cells, which is essential for high-throughput applications [133]. However, maintaining high accuracy across extended sorting ranges requires precise calibration and sophisticated system design. These improvements highlight the various capabilities and inherent trade-offs of each technology, with microfluidic integration providing scalable, cost-effective solutions suitable for a wide range of biomedical applications, albeit seeking meticulous calibration to maintain precision.

By integrating these advanced techniques, the limits of traditional cell sorting have been expanded, enhancing both the accuracy and efficiency of the process. Each method presents unique advantages: holographic techniques deliver superior spatial resolution, whereas microfluidic systems provide scalable and low-cost implementations. However, both approaches must be carefully calibrated to maintain precise control over extended ranges. These studies, therefore, highlight a broad spectrum of capabilities and limitations, demonstrating that while optical tweezers combined with microfluidics, holography, and fluorescence considerably advance cell sorting, each method must be selected based on the specific requirements of the application.

Optical tweezers have significantly contributed to cell manipulation and sorting by allowing precise and non-invasive cell handling. These advances have expanded their applicability in biomedical research, diagnostics, and treatments, making them vital in contemporary cell biology and biophysics. Through these capabilities, optical tweezers have become indispensable in advancing our understanding of cellular processes, enabling high-precision experiments that elucidate the complexities of cellular dynamics and contribute to the development of advanced biomedical applications.

### Pathogen-host interactions

Optical tweezers have emerged as a substantial technique in studying pathogen-host interactions. This allows researchers to exert precise forces on individual cells, bacteria, and even subcellular components, facilitating a detailed examination of these interactions' mechanic and dynamic aspects. One can observe and quantify the forces exerted during the attachment and invasion of pathogens into host cells by employing optical tweezers, providing insights into the mechanical properties and the molecular mechanisms underpinning these processes. This capability is particularly crucial for understanding the complexities of pathogen adherence, the efficacy of immune responses, and the development of novel therapeutic strategies. For example, Tam *et al*. used optical tweezers combined with spinning disk confocal microscopy to study the dynamic interactions between phagocytic cells and pathogens, enabling precise control over pathogen placement and facilitating detailed observations of immune responses [134]. Later, they utilized optical traps to manipulate live pathogens like *Candida albicans* and *Aspergillus fumigatus*, positioning these pathogens near macrophages to observe the early stages of phagocytosis with high spatial and temporal resolution [135]. This approach offered new insights into the initial interactions between pathogens and immune cells. Kemper *et al*. integrated digital holographic microscopy with holographic optical tweezers for manipulating bacteria and real-time monitoring of infection processes at the single-cell level [136]. This enabled the precise alignment of bacterial cells on host surfaces, which is crucial for studying infection mechanisms. Similarly, Crick *et al*. used optical tweezers to quantify the interactions between *Plasmodium falciparum* merozoites and erythrocytes, revealing the adhesive forces involved in malaria parasite invasion [137].

Moreover, optical tweezers combined with various techniques were employed to study interactions between plant cell organelles [138], bacterial adhesion and infection processes [139], forces between T-cells and antigen-presenting cells [140], single HIV-1virion analysis [141], binding forces between lymphocyte function-associated antigen-1 on natural killer cells and their monoclonal antibodies [142].

Optical tweezers have become a crucial tool in studying pathogen-host interactions, enabling precise manipulation and real-time observation of cellular and molecular processes. The application of optical tweezers in this context has thus expanded our comprehension of the biophysical aspects of infections and the host's defensive mechanisms, offering a nuanced perspective that complements traditional biochemical and genetic approaches. These advancements have significantly enhanced our understanding of infection mechanisms, immune responses, and cellular biomechanics, highlighting the technique's indispensable role in biomedical research.

### In-vitro fertilization

Optical tweezers have been shown promising applications in in-vitro fertilization (IVF). As a non-invasive and contactless micromanipulation method, optical tweezers allow precise handling of gametes and embryos, which makes it a crucial tool for IVF.

Optical tweezers are successfully employed to manipulate sperm and oocytes with exceptional accuracy. A notable study demonstrated the use of a combined UV-laser microbeam and optical tweezer trap for performing laser zona drilling and sub-zonal insemination in cattle [143]. The UV-laser microbeam was used to drill a small channel in the zona pellucida, the outer layer of the oocyte, through which a single sperm was transported using optical tweezers. This method facilitated the direct insertion of the sperm into the perivitelline space and brought it into close contact with the oolemma, thereby enhancing the chances of sperm-oocyte fusion. This technique is advantageous as it eliminates the need for mechanical microtools, reducing potential damage to the gametes and ensuring a sterile environment for manipulation [143].

## CHALLENGES AND FUTURE DIRECTIONS IN OPTICAL TWEEZERS APPLICATIONS

Optical tweezers have emerged as a transformational technique in biomedical research, allowing for precise, non-invasive manipulation of cells, macromolecules, and individual molecules. Their ability to apply tiny forces allows for the investigation of complex biological systems such as cell mechanical characteristics, protein-DNA interactions, and molecular motors. Despite the aforementioned advantages, optical tweezers experience a number of limitations that must be addressed for their effective utilization, particularly at the cellular and molecular levels.

One major concern is the photothermal effect of laser, which results from light absorption and causes localized heating [144]. This effect can induce unwanted damage to the biological specimens by producing protein denaturation or changing biochemical processes. This issue is important to consider when optical tweezers are applied to small particles during lengthy investigations using higher laser powers [145]. To overcome this issue, infrared lasers (1064 nm) are recommended for use in biological applications as they are less absorbed by biological tissues [9]. Furthermore, reducing laser power and adopting temperature-controlled fluidic chambers can reduce heating while keeping sample integrity [146]. Real-time temperature monitoring technologies, such as fluorescence-based thermometry, can also be used to regulate trapping conditions and prevent overheating. Additionally, in biological applications, optical tweezers can be most efficient only in a small force range (pN to nN) [8], which limits its application to stiffer materials or bigger deformations that require higher forces. Similar techniques, such as atomic force microscopy (AFM) or micropipette aspiration, may be more appropriate in such cases.

Furthermore, optical tweezers are sensitive to environmental conditions such as temperature changes and optical aberrations, which can affect trap stability and measurement precision. Advances such as adaptive optics, which correct for aberrations in real-time, and experiments under thermally controlled environment conditions can reduce these issues. Integrating optical tweezers with complementing techniques such as fluorescence microscopy and microfluidics can improve their capabilities and enable more detailed analysis.

## CONCLUSION

While optical tweezers provide exceptional precision for manipulating biological systems at the cellular and molecular levels, they have certain limitations. Issues such as the photothermal effect, reduced efficiency for trapping small particles, and a restricted force range must be addressed. However, ongoing advancements in laser technology, trap designs, and integration with complementary techniques are expanding their capabilities, solidifying optical tweezers as an essential tool across fields from molecular biophysics to biomedical diagnostics.
